# *Strongyloides stercoralis* Infection and Spirometry-Defined Airway Obstruction in Children with Sickle Cell Disease: A Species-Specific Multicentre Cross-Sectional Study in French Guiana

**DOI:** 10.3390/pathogens15080867

**Published:** 2026-08-19

**Authors:** Gabriel Bafunyembaka, Chimene Maniassom, Philbert Furero, Arriel Makembi, Pascal Kuamba, Narcisse Elenga

**Affiliations:** 1 Department of Pediatrics, Franck Joly Hospital, Western French Guiana Hospital Center (CHOG), 1465 Boulevard de la Liberté, 97320 Saint-Laurent-du-Maroni, French Guiana, France; p.furero@chu-guyane.fr (P.F.); p.kuamba@chu-guyane.fr (P.K.); 2 Department of Pediatrics, General Reference Hospital of Bukavu, Bukavu, Democratic Republic of the Congo; 3 Department of Pediatrics, Cayenne Hospital, Centre Hospitalier Andrée Rosemon, 3 Avenue Alexis Blaise, 97300 Cayenne, French Guiana, France; maniassomc@ch-cayenne.fr (C.M.); elengafr@yahoo.fr (N.E.); 4 Department of Internal Medicine, Franck Joly Hospital, Western French Guiana Hospital Center (CHOG), 1465 Boulevard de la Liberté, 97320 Saint-Laurent-du-Maroni, French Guiana, France; a.makembi@chu-guyane.fr

**Keywords:** *Strongyloides stercoralis*, sickle cell disease, children, airway obstruction, spirometry, epidemiology, French Guiana

## Abstract

Background/Objectives: *Strongyloides stercoralis* may persist through autoinfection and is clinically important before corticosteroid exposure, but its relationship with airway obstruction in children with sickle cell disease (SCD) is uncertain. We examined whether routine-detected *S. stercoralis* infection was associated with spirometry-defined airway obstruction in children with SCD in French Guiana. Methods: This multicentre cross-sectional secondary analysis used an availability-based sample from a routine respiratory screening database. Eligible children aged 5–17 years had SCD, spirometry, and stool parasitology documented during routine care. The primary outcome was FEV1/FVC < 80%. Logistic regression adjusted for age, sex, genotype, hydroxyurea treatment, previous acute chest syndrome, and atopy. Results: Stool parasitology was available for 160 children; 26 (16.2%) had *S. stercoralis* detected. Among 138 children with complete spirometry and covariate data, 45 (32.6%) had obstruction. *S. stercoralis* was not associated with obstruction in univariable analysis (OR 0.75, 95% CI 0.30–1.91) or after adjustment (adjusted OR 1.02, 95% CI 0.37–2.86; *p* = 0.964). Male sex was independently associated with obstruction (adjusted OR 2.32, 95% CI 1.11–4.83). Conclusions: Routine-detected *S. stercoralis* infection was not an independent correlate of airway obstruction. The wide confidence interval, limited exposure ascertainment, and small infected subgroup mean that clinically important protective or harmful associations cannot be excluded.

## 1. Introduction

Sickle cell disease (SCD) is an inherited haemoglobin disorder associated with substantial paediatric respiratory morbidity. Acute chest syndrome, recurrent wheezing, exercise limitation, and chronic lung-function abnormalities contribute to hospitalisation and long-term pulmonary risk [1,2,3]. Asthma and asthma-like symptoms may further increase acute chest syndrome and healthcare use, although airflow obstruction in SCD may also reflect airway hyperresponsiveness, recurrent infection, systemic inflammation, endothelial dysfunction, or pulmonary vascular disease [4,5,6,7,8,9,10].

Helminth infections may influence respiratory health through type 2 inflammation, eosinophilia, and regulatory immune pathways, but reported associations with asthma vary according to parasite species, intensity, chronicity, host background, and diagnostic method [11,12,13,14,15,16]. *Strongyloides stercoralis* is particularly relevant because chronic infection can persist through autoinfection and corticosteroid exposure may precipitate severe hyperinfection [17,18,19,20].

French Guiana combines a high burden of SCD with tropical parasitic exposure. Respiratory symptoms in this setting require careful phenotyping because attributing airway disease to a single infectious exposure may be misleading, while failure to recognize strongyloidiasis before corticosteroid treatment may have serious consequences.

A related multicentre study from the broader French Guianese paediatric SCD cohort evaluated intestinal parasitic infection as a composite exposure [21]. The present analysis addresses the narrower question of whether routine-detected *S. stercoralis* is associated with objective airway obstruction. The mechanistic hypothesis was that chronic infection might contribute to type 2/eosinophilic airway inflammation and airway hyperresponsiveness. Separately, irrespective of any respiratory association, identifying strongyloidiasis has a clinical safety rationale because corticosteroid exposure may precipitate hyperinfection. We therefore hypothesised that detected *S. stercoralis* infection might be associated with an obstructive respiratory phenotype.

## 2. Materials and Methods

### 2.1. Study Design and Setting

We conducted an exploratory multicentre cross-sectional secondary analysis among children with SCD followed at the public hospitals of Cayenne, Kourou, and Saint-Laurent-du-Maroni. The analysis was embedded in a respiratory screening programme integrated into routine paediatric haematology care. It was not a population-based random sample: the analytical sample comprised all eligible children in the screening database for whom both spirometry and stool parasitology were documented and is therefore best considered an availability-based or convenience sample. Retrospective clinical data were obtained from medical records, hospitalisation reports, and hospital information systems. The clinical follow-up window covered the 12 months preceding respiratory assessment.

### 2.2. Participants

Children were eligible if they were 5–17 years old, had confirmed SCD based on haemoglobin electrophoresis or molecular testing, and had a respiratory evaluation including spirometry together with a stool parasitology result documented during routine care. Children were not selected because of respiratory or gastrointestinal symptoms. However, the indication for each stool examination was not consistently recorded, so the database could not reliably distinguish systematic screening from clinician-directed testing based on symptoms or exposure risk. HbSS and HbSC genotypes were represented. Children unable to perform acceptable spirometry or with incomplete data precluding primary outcome assessment were excluded from the corresponding analyses.

### 2.3. Respiratory Assessment and Outcomes

Spirometry was performed according to ATS/ERS recommendations, with bronchodilator testing after inhaled salbutamol. A result was considered non-interpretable when the child did not produce technically acceptable and sufficiently repeatable forced expiratory manoeuvres, or when an incomplete FEV1 and/or FVC recording precluded calculation or reliable interpretation of the FEV1/FVC ratio. The primary outcome was an obstructive respiratory phenotype, defined pragmatically as FEV1/FVC < 80% when lower-limit-of-normal values were unavailable. This threshold was selected instead of the adult fixed ratio of 0.70 because normal FEV1/FVC ratios are higher and age-dependent in children; consequently, the adult 0.70 cutoff can miss clinically relevant airflow limitation in paediatric populations. Age-, sex-, height-, and ancestry-adjusted lower limits of normal remain the preferred standard, and the fixed 80% threshold was used only because the variables required to calculate GLI z-scores were not consistently available across centres [22,23]. This binary outcome could be applied consistently across the multicentre dataset and maximised the analysable sample. It identifies airflow obstruction but does not establish reversibility or asthma. Bronchodilator reversibility was defined as an increase in FEV1 of at least 12%. Physician-diagnosed asthma with bronchodilator reversibility and the mutually exclusive descriptive categories normal, obstructive, and unclassified were secondary outcomes. The mismatch between a type 2/airway-hyperresponsiveness hypothesis and a non-reversibility-based primary outcome was considered when interpreting the findings.

### 2.4. Parasitological Assessment

Stool parasitology was performed during routine care and was not a research-specific procedure. Children were classified according to documented *S. stercoralis* status. Hospital laboratories used direct microscopy and concentration techniques according to local practice. The clinical indication for testing was not consistently available. In most cases, one stool sample was examined; systematic serology, PCR, Baermann technique, and agar plate culture were not routinely available. Consequently, a negative result indicated no parasite detected in the available routine-care sample rather than definitive exclusion of infection.

### 2.5. Covariates

Covariates included age, sex, place of residence, SCD genotype, hydroxyurea treatment, previous acute chest syndrome, and atopy defined as at least one positive skin-prick test. Rural residence was available as a limited contextual proxy, but direct measures of household income, housing quality, sanitation, overcrowding, migration history, and parental education were unavailable. These variables were selected according to clinical relevance and availability.

### 2.6. Statistical Analysis

Continuous variables were summarised using means and standard deviations or medians and interquartile ranges, as appropriate. Categorical variables were reported as counts and percentages. Between-group comparisons used the Mann–Whitney U test, chi-square test, or Fisher’s exact test. Logistic regression estimated the association between *S. stercoralis* and obstruction. Variables with *p* < 0.20 in univariable analyses and clinically relevant covariates identified a priori were considered for adjustment. The final model included *S. stercoralis* status, age, sex, genotype, hydroxyurea treatment, previous acute chest syndrome, and atopy. Rural residence met the screening threshold but was not added because only 45 primary-outcome events and 26 exposed children were available; adding another parameter to an already exploratory model was judged likely to worsen overfitting and estimate instability. Results are presented as odds ratios (ORs) with 95% confidence intervals (CIs). Statistical significance was set at *p* < 0.05. No prospective sample-size calculation was performed because the analysis used all eligible routine-care records. An approximate post hoc two-group calculation (two-sided α = 0.05, 80% power, exposure prevalence about 16%, and outcome prevalence 32.6%) indicated that the available sample could reliably detect only large effects, approximately OR ≤ 0.17 or OR ≥ 3.8. Reporting followed the STROBE statement [24]; the completed checklist is provided as Supplementary File S1.

### 2.7. Patient and Public Involvement

Patients and members of the public were not involved in the design, conduct, reporting, or dissemination planning of this secondary analysis.

### 2.8. Ethics and Data Protection

This study used only pseudonymised clinical and laboratory data collected during routine care; no additional examination, intervention, or biological sampling was performed. Under Article R1121-1, II(3), of the French Public Health Code, this secondary use of existing health data is not classified as research involving human participants and did not require approval by a Comité de Protection des Personnes. Data processing followed the applicable French data-protection framework and MR-004 methodology. Research-specific consent was not required; parents or legal guardians were informed of secondary data use and their right to object under institutional procedures.

## 3. Results

### 3.1. Study Population

**Figure 1:**
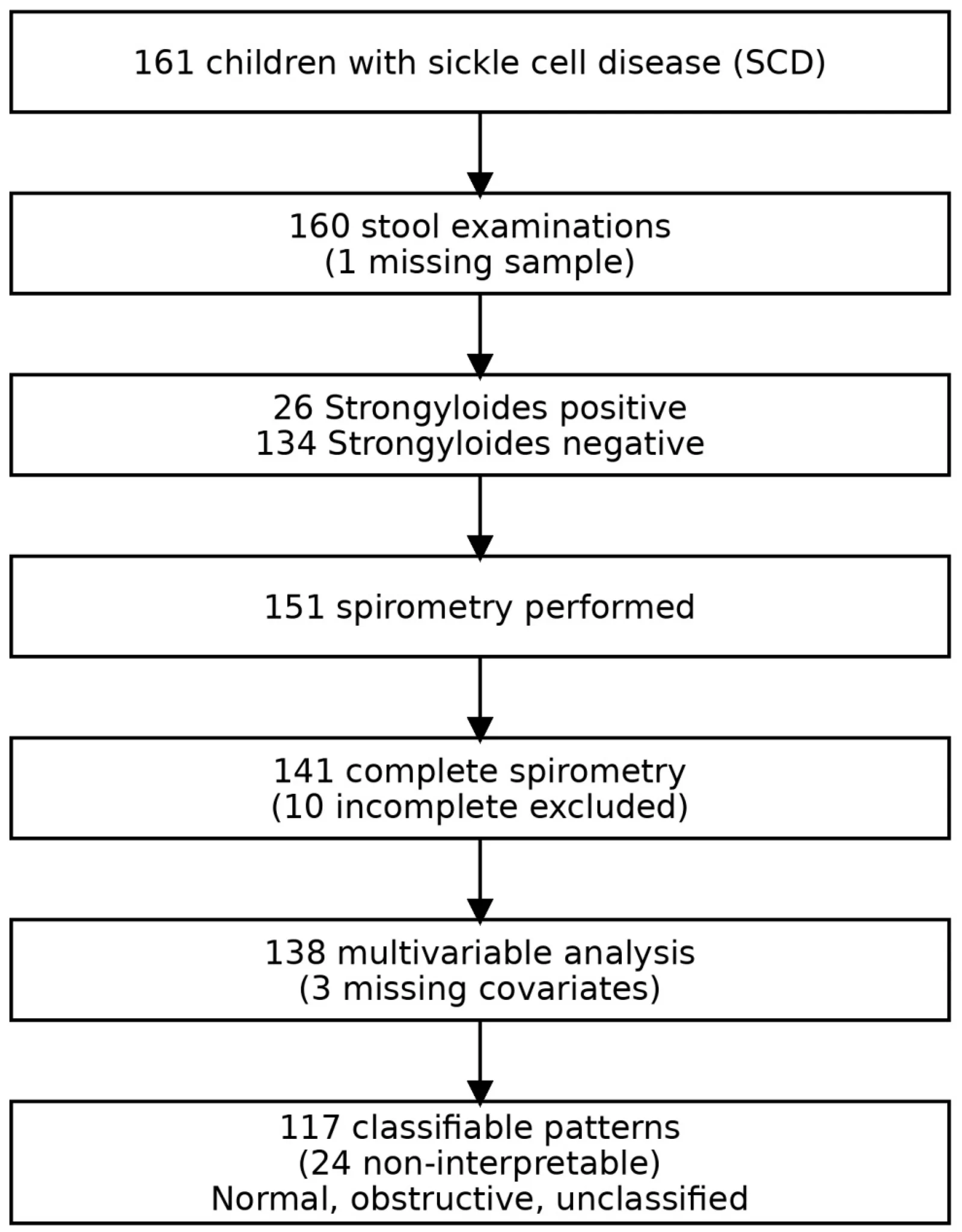
Flowchart of participant selection and inclusion in respiratory and parasitological analyses. A total of 161 children with SCD underwent respiratory assessment; stool parasitology was available for 160, spirometry was attempted in 151, and complete spirometry data were available for 141. The ten exclusions between attempted and complete spirometry reflected incomplete usable measurements; the retrospective database did not allow these exclusions to be separated reliably into failed acceptable manoeuvres versus other incomplete records or to be stratified by age. Among the 141 complete records, 117 yielded interpretable categorical patterns, and 24 were non-interpretable; 138 children had complete covariates for multivariable analysis.

A total of 161 children with SCD underwent respiratory assessment. Stool parasitology was available for 160 children, of whom 26 (16.2%) had *S. stercoralis* detected and 134 (83.8%) had no detected infection. Spirometry was attempted in 151 children. Ten were excluded before the complete spirometry dataset because the available record did not contain a complete set of usable spirometric measurements. The retrospective database did not preserve sufficiently granular reasons to determine how many of these ten exclusions were specifically due to inability to perform an acceptable manoeuvre rather than incomplete recording, nor did it permit reliable stratification of these exclusions by age band. Complete spirometry data were available for 141 children; 138 also had complete covariate data for multivariable analysis (Figure 1). The primary binary outcome, FEV1/FVC < 80%, was present in 45 of these 138 children (32.6%).

Using the separate descriptive classification scheme, 117 children had interpretable spirometric patterns: Notably, 45 (38.5%) were obstructive, 40 (34.2%) were normal, and 32 (27.4%) were unclassified or borderline. The unclassified/borderline category comprised technically usable recordings that did not satisfy the predefined criteria for either a normal or obstructive pattern; it was distinct from the 24 non-interpretable results in which technical quality or completeness was insufficient for categorical interpretation. The identical count of 45 obstructive cases under the two schemes is coincidental: the binary primary outcome used *n* = 138 and an FEV1/FVC threshold, whereas the categorical pattern analysis used *n* = 117 after excluding non-interpretable results. Physician-diagnosed asthma with bronchodilator reversibility was identified in 95 children. Baseline characteristics according to *S. stercoralis* status are presented in Table 1.

### 3.2. Association with Airway Obstruction

*S. stercoralis* infection was not associated with obstruction in univariable analysis (OR 0.75, 95% CI 0.30–1.91; *p* = 0.550). Male sex was associated with obstruction (OR 2.51, 95% CI 1.26–5.00; *p* = 0.009), whereas atopy showed a non-significant trend (OR 2.86, 95% CI 0.94–8.74; *p* = 0.065; Table S1).

After adjustment, *S. stercoralis* remained unassociated with obstruction (adjusted OR 1.02, 95% CI 0.37–2.86; *p* = 0.964). Male sex was the only independently associated factor (adjusted OR 2.32, 95% CI 1.11–4.83; *p* = 0.025). Age, genotype, hydroxyurea treatment, previous acute chest syndrome, and atopy were not statistically significant (Table 2).

### 3.3. Secondary Descriptive Findings

Physician-diagnosed asthma with bronchodilator reversibility was similarly frequent in children with and without detected *S. stercoralis* infection (53.8% vs. 60.4%; *p* = 0.52). Overall intestinal parasitic exposure was not associated with obstruction or physician-diagnosed asthma with bronchodilator reversibility in descriptive analyses; these composite-exposure findings are not the primary focus of the present species-specific report.

## 4. Discussion

In this multicentre, paediatric SCD cohort, routine-detected *S. stercoralis* infection was not associated with spirometry-defined airway obstruction or physician-diagnosed asthma with bronchodilator reversibility. The crude estimate suggested a weak protective trend (OR 0.75), whereas adjustment shifted the estimate slightly above the null (aOR 1.02). This change in direction is most plausibly explained by confounding structure and small-sample instability rather than a biologically meaningful reversal. Importantly, the adjusted 95% CI (0.37–2.86) is compatible with a substantial reduction or nearly a threefold increase in the odds of obstruction; therefore, the study cannot rule out clinically important benefit or harm.

The null point estimate does not establish the absence of respiratory relevance. Single-sample stool microscopy has limited sensitivity for chronic or low-intensity infection, and nondifferential exposure misclassification would tend to attenuate associations. The available sample was also capable of detecting only large effects. The present finding is consistent with the heterogeneous literature reviewed by Arrais et al. [16], in which associations between intestinal helminths and asthma or allergic disease varied by organism, setting, and diagnostic method. Thus, our results indicate only that routinely detected infection was not a major independent correlate of obstruction under the diagnostic conditions used.

This *S. stercoralis*-focused analysis used the same broader screening infrastructure as the previously published composite-parasite study [21]. Of the 161 children in the present source population, 140 (87.0%) were also represented in the companion cohort. The present report is therefore a reanalysis of a heavily overlapping population rather than an independent replication. Its distinct contribution is the species-specific exposure definition, the binary spirometric primary outcome, and the dedicated multivariable model. This overlap is now stated explicitly so that novelty is interpreted appropriately.

The mechanistic and clinical-safety arguments should be separated. Mechanistically, a chronic Th2/eosinophilic pathway would be expected to manifest most directly as reversible airway hyperresponsiveness. Our primary FEV1/FVC < 80% outcome captures airflow obstruction but does not distinguish reversible from fixed obstruction; the absence of association with both the primary outcome and the secondary reversible-asthma phenotype does not definitively test the proposed immune mechanism. This outcome-mechanism mismatch is a specific limitation.

Independently of the mechanistic hypothesis, strongyloidiasis remains clinically important because systemic corticosteroids can precipitate hyperinfection [17,18,19,20]. Children with SCD and respiratory symptoms may receive corticosteroids for presumed asthma exacerbations. Accordingly, exposure-risk assessment and sensitive testing or presumptive treatment in selected patients before immunosuppression remain safety priorities regardless of whether *S. stercoralis* is associated with airway obstruction. Male sex was the only factor independently associated with obstruction, consistent with reported sex-related differences in childhood wheeze and airflow limitation [25]. Atopy showed a non-significant trend, but the study was not powered to evaluate interactions among atopy, helminth infection, and SCD genotype.

Strengths include multicentre inclusion, objective spirometry with bronchodilator testing, and a focused analysis of a neglected infection in a vulnerable population. The observed *S. stercoralis* detection rate of 16.2% is higher than modelled global general-population estimates, but comparisons are limited by major differences in geography, age, sampling, and diagnostic sensitivity [26]. Likewise, no contemporaneous non-SCD paediatric control group using the same spirometry protocol was available; the 32.6% obstruction prevalence should therefore not be interpreted as an SCD-attributable excess. Social disadvantage may influence both parasitic exposure and respiratory health. Rural residence was the only available contextual proxy and showed a univariable trend, but detailed measures of housing, sanitation, income, overcrowding, and education were unavailable. Spirometry feasibility is age-dependent, particularly in younger children [27]. Ten children were lost between attempted and complete spirometry, but the retrospective database did not record whether each loss resulted from inability to achieve an acceptable manoeuvre or from another form of incomplete documentation, and age-band-specific exclusion counts were unavailable. Therefore, preferential retention of older or more technically compliant children cannot be excluded, and adjustment for age within the regression model would not eliminate this selection mechanism. Other limitations include the cross-sectional design, availability-based sampling, uncertain indication for stool testing, only 26 infected children, mostly single-sample microscopy, absence of systematic serology or molecular testing, use of a fixed FEV1/FVC threshold rather than age-adjusted z-scores, exclusion of rural residence from the final model to limit overfitting, and incomplete eosinophil, IgE, FeNO, asthma-control, and corticosteroid-exposure data. These limitations reduced power and may have obscured parasite-specific or immune-mediated associations.

## 5. Conclusions

Routine-detected *S. stercoralis* infection was not independently associated with spirometry-defined airway obstruction in this availability-based cohort of children with SCD in French Guiana. However, the wide confidence interval, limited diagnostic sensitivity, and small exposed subgroup mean that clinically important protective or harmful associations cannot be excluded. These findings are hypothesis-generating and support prospective studies using repeated sensitive parasitological testing, standardised lung-function definitions, and immunological assessment.

## Figures and Tables

**Table 1:** Baseline characteristics according to *Strongyloides stercoralis* status.

Variable	*S. stercoralis* Not Detected (*n* = 134)	*S. stercoralis* Detected (*n* = 26)	*p* Value
Age, years, mean ± SD	10.1 ± 3.5	9.8 ± 2.9	0.68
Male sex, *n* (%)	65 (48.5)	12 (46.2)	0.84
Female sex, *n* (%)	69 (51.5)	14 (53.8)	0.84
HbSS genotype, *n* (%)	95 (70.9)	21 (80.8)	0.32
HbSC genotype, *n* (%)	39 (29.1)	5 (19.2)	0.32
Rural residence, *n* (%)	103 (76.9)	16 (61.5)	0.09
Hydroxyurea treatment, *n* (%)	99 (73.9)	22 (84.6)	0.23
Previous acute chest syndrome, *n* (%)	52 (38.8)	7 (26.9)	0.24
Physician-diagnosed asthma with bronchodilator reversibility, *n* (%)	81 (60.4)	14 (53.8)	0.52
Normal spirometry pattern, *n* (%)	35 (33.3)	5 (41.7)	0.32
Obstructive spirometry pattern, *n* (%)	40 (38.1)	5 (41.7)	0.52
Unclassified spirometry pattern, *n* (%)	30 (28.6)	2 (16.7)	1.00

Spirometry pattern percentages were calculated among children with classifiable results (*n* = 117). SD, standard deviation.

**Table 2:** Multivariable logistic regression for spirometry-defined airway obstruction (FEV1/FVC < 80%).

Variable	Adjusted OR	95% CI	*p* Value
*S. stercoralis* detected (yes vs. no)	1.02	0.37–2.86	0.964
Age, per year	0.99	0.89–1.10	0.862
Male sex (vs. female)	2.32	1.11–4.83	0.025
HbSS genotype (vs. HbSC)	1.63	0.62–4.28	0.321
Hydroxyurea treatment (yes vs. no)	0.87	0.31–2.44	0.791
Previous acute chest syndrome (yes vs. no)	1.41	0.64–3.10	0.389
Atopy (≥1 positive skin-prick test)	2.12	0.63–7.14	0.224

Regression population: *n* = 138. CI, confidence interval; OR, odds ratio.

## Data Availability

The data are not publicly available because they contain sensitive clinical information from a small paediatric population. De-identified data may be made available by the corresponding author upon reasonable request, subject to institutional authorisation and applicable legal and data-protection requirements.

## Supplementary Materials

The following supporting information can be downloaded at https://www.mdpi.com/article/10.3390/pathogens15080867/s1, Table S1. Univariable logistic regression analysis for spirometry-defined airway obstruction (FEV1/FVC < 80%); Supplementary File S1. STROBE Statement—Checklist of items that should be included in reports of cross-sectional studies.
