# Supplementary material for: Strongyloides stercoralis Infection and Spirometry-Defined Airway Obstruction in Children with Sickle Cell Disease: A Species-Specific Multicentre Cross-Sectional Study in French Guiana
Source: Pathogens. 2026 Aug 19;15(8):867. doi: 10.3390/pathogens15080867 (PMC13516418; doi:10.3390/pathogens15080867)
Supplement: Supplementary file 1 [file pathogens-15-00867-s001.zip › Supplementary File S1.pdf]

STROBE Statement—Checklist of items that should be included in reports of cross-sectional studies

Manuscript: Strongyloides stercoralis Infection and Spirometry-Defined Airway Obstruction in Children with Sickle Cell Disease: A Species-Specific Multicentre Study in French Guiana

Section/topic	Item	STROBE recommendation	Location in manuscript / assessment
**Title and abstract**	1(a)	Indicate the study's design with a commonly used term in the title or abstract.	Abstract, p. 1: "multicentre cross-sectional secondary analysis." The title states "multicentre study" but does not explicitly include "cross-sectional."
Title and abstract	1(b)	Provide in the abstract an informative and balanced summary of what was done and what was found.	Abstract, p. 1: background/objective, methods, sample sizes, crude and adjusted estimates, and cautious conclusion.
**Introduction—Background/rationale**	2	Explain the scientific background and rationale for the investigation being reported.	Introduction, pp. 2–3: respiratory morbidity in SCD, possible helminth–airway mechanisms, relevance of S. stercoralis, and the French Guiana context.
**Introduction—Objectives**	3	State specific objectives, including any prespecified hypotheses.	Introduction, p. 2: species-specific objective and hypothesis that S. stercoralis might be associated with an obstructive respiratory phenotype.
**Methods—Study design**	4	Present key elements of study design early in the paper.	Methods 2.1, p. 2: exploratory multicentre cross-sectional study embedded in routine respiratory screening; retrospective clinical data and a 12-month clinical follow-up window.
**Methods—Setting**	5	Describe the setting, locations, and relevant dates, including periods of recruitment, exposure, follow-up, and data collection.	Methods 2.1, p. 2: public hospitals of Cayenne, Kourou, and Saint-Laurent-du-Maroni; routine paediatric haematology care; 12 months preceding respiratory assessment. Exact calendar recruitment/data-collection dates are not reported.
**Methods—Participants**	6(a)	Give eligibility criteria and the sources and methods of selection of participants.	Methods 2.2, pp. 2–3: children aged 5–17 years with confirmed SCD who underwent spirometry and stool parasitology; exclusions for unacceptable/incomplete spirometry or incomplete outcome data.
**Methods—Variables**	7	Clearly define all outcomes, exposures, predictors, potential confounders, and effect modifiers. Give diagnostic criteria, if applicable.	Methods 2.3–2.5, p. 3: obstruction defined as FEV_1/FVC <80%; bronchodilator reversibility ≥12%; documented S. stercoralis status; age, sex, residence, genotype, hydroxyurea, previous ACS, and atopy.
**Methods—Data sources/measurement**	8	For each variable of interest, give sources of data and details of methods of assessment. Describe comparability of assessment methods if there is more than one group.	Methods 2.1–2.5, pp. 2–3: medical records/hospital systems; ATS/ERS spirometry with salbutamol; routine stool microscopy and concentration methods; atopy by skin-prick testing. Local laboratory practice is described, but centre-level comparability is not detailed.
**Methods—Bias**	9	Describe any efforts to address potential sources of bias.	Methods 2.4 and Discussion, pp. 3 and 6: negative stool results interpreted cautiously; limitations from single-sample microscopy and exposure misclassification are acknowledged. No formal bias-control procedure is reported.
**Methods—Study size**	10	Explain how the study size was arrived at.	Not reported. The study used the available respiratory-screening cohort; no formal sample-size calculation is presented.
**Methods—Quantitative variables**	11	Explain how quantitative variables were handled in the analyses. If applicable, describe which groupings were chosen and why.	Methods 2.3, 2.5, and 2.6, p. 3: age analysed per year; FEV_1/FVC dichotomised at <80%; continuous variables summarised by mean/SD or median/IQR. Rationale for the fixed threshold is provided pragmatically because LLN values were unavailable.
**Methods—Statistical methods**	12(a)	Describe all statistical methods, including those used to control for confounding.	Methods 2.6, p. 3: Mann–Whitney U, chi-square/Fisher tests, univariable and multivariable logistic regression; clinically relevant covariates and variables with p<0.20 considered; final adjustment set specified.
Methods—Statistical methods	12(b)	Describe any methods used to examine subgroups and interactions.	Not reported; no subgroup or interaction analyses were described.
Methods—Statistical methods	12(c)	Explain how missing data were addressed.	Partially reported in participant flow (pp. 3 and 5): incomplete spirometry and missing covariates were excluded from relevant analyses. No imputation method was used or described.
Methods—Statistical methods	12(d)	If applicable, describe analytical methods taking account of sampling strategy.	Not applicable; no complex sampling strategy was reported.
Methods—Statistical methods	12(e)	Describe any sensitivity analyses.	Not reported.
**Results—Participants**	13(a)	Report numbers of individuals at each stage of the study.	Results 3.1 and Figure 1, pp. 3 and 5: 161 assessed; stool parasitology 160; spirometry 151; complete spirometry 141; multivariable analysis 138; classifiable patterns 117.
Results—Participants	13(b)	Give reasons for non-participation at each stage.	Figure 1, p. 5: one missing stool sample, ten incomplete spirometry tests, three missing covariates, and 24 non-interpretable patterns. Further reasons are not detailed.
Results—Participants	13(c)	Consider use of a flow diagram.	Figure 1, p. 5 provides a participant flowchart.
**Results—Descriptive data**	14(a)	Give characteristics of study participants and information on exposures and potential confounders.	Table 1, p. 4: age, sex, genotype, residence, hydroxyurea, previous ACS, asthma with reversibility, and spirometry pattern by S. stercoralis status.
Results—Descriptive data	14(b)	Indicate the number of participants with missing data for each variable of interest.	Partially reported: overall counts for missing stool sample, incomplete spirometry, and missing covariates are shown in Figure 1; variable-specific missingness is not fully tabulated.
**Results—Outcome data**	15	Report numbers of outcome events or summary measures.	Results 3.1, pp. 3–4: 45 obstructive patterns among 117 classifiable spirometry results; abstract reports 45/138 for the regression population, which should be checked for consistency.
**Results—Main results**	16(a)	Give unadjusted estimates and, if applicable, confounder-adjusted estimates and their precision. Make clear which confounders were adjusted for and why.	Results 3.2, p. 4; Supplementary Table S1 and Table 2: crude and adjusted ORs with 95% CIs and p values; final covariate set described in Methods 2.6.
Results—Main results	16(b)	Report category boundaries when continuous variables were categorized.	Methods 2.3, p. 3: airway obstruction defined as FEV_1/FVC <80%; bronchodilator reversibility defined as ≥12% increase in FEV_1.
Results—Main results	16(c)	If relevant, consider translating estimates of relative risk into absolute risk for a meaningful time period.	Not applicable to this cross-sectional logistic-regression analysis.
**Results—Other analyses**	17	Report other analyses performed, such as subgroup, interaction, and sensitivity analyses.	No subgroup, interaction, or sensitivity analyses were reported. Secondary descriptive findings are provided in Results 3.3, pp. 4–5.
**Discussion—Key results**	18	Summarise key results with reference to study objectives.	Discussion, pp. 5–6: no association between routinely detected S. stercoralis and obstruction/asthma with reversibility; male sex independently associated with obstruction.
**Discussion—Limitations**	19	Discuss limitations, taking into account sources of potential bias or imprecision. Discuss direction and	Discussion, p. 6: cross-sectional design, only 26 infected children, single-sample microscopy, absent

Section/topic	Item	STROBE recommendation	Location in manuscript / assessment
		magnitude of potential bias.	serology/PCR, fixed FEV_1/FVC threshold, and incomplete biomarkers/clinical data; exposure misclassification likely attenuated associations.
**Discussion—Interpretation**	20	Give a cautious overall interpretation considering objectives, limitations, multiplicity, similar studies, and other relevant evidence.	Discussion and Conclusions, p. 6: null finding interpreted cautiously as hypothesis-generating; clinical importance before corticosteroids maintained; overlap with the broader cohort acknowledged.
**Discussion—Generalisability**	21	Discuss the generalisability (external validity) of the study results.	Partially addressed through the multicentre French Guiana context and limitations, pp. 5–6. A separate explicit discussion of generalisability is not provided.
**Other information—Funding**	22	Give the source of funding and the role of the funders.	Funding statement, p. 6: “This research received no external funding.”

Abbreviations: ACS, acute chest syndrome; CI, confidence interval; FEV_1, forced expiratory volume in 1 second; FVC, forced vital capacity; LLN, lower limit of normal; OR, odds ratio; SCD, sickle cell disease.

Completion note: Locations were assigned from the submitted 8-page Pathogens manuscript. Entries marked “not reported” or “partially reported” identify information that is absent or incomplete in the current manuscript and should not be treated as newly supplied data.
