# Supplementary material for: Strongyloides stercoralis Infection and Spirometry-Defined Airway Obstruction in Children with Sickle Cell Disease: A Species-Specific Multicentre Cross-Sectional Study in French Guiana
Source: Pathogens. 2026 Aug 19;15(8):867. doi: 10.3390/pathogens15080867 (PMC13516418; doi:10.3390/pathogens15080867)
Supplement: Supplementary file 1 [file pathogens-15-00867-s001.zip › Supplementary Table S1.pdf]

Table S1. Univariable logistic regression analysis for spirometry-defined airway obstruction (FEV_1/FVC <80%).

Variable	Crude OR	95% CI	p-value
Strongyloides stercoralis infection (positive vs. negative)	0.75	0.30 – 1.91	0.550
Age (per year)	0.99	0.89 – 1.10	0.862
Male sex (vs. female)	2.51	1.26 – 5.00	0.009
HbSS genotype (vs. HbSC)	1.45	0.58 – 3.60	0.420
Hydroxyurea treatment (yes vs. no)	0.92	0.35 – 2.41	0.870
Previous acute chest syndrome (yes vs. no)	1.38	0.66 – 2.90	0.380
Atopy (≥1 positive skin-prick test)	2.86	0.94 – 8.74	0.065

CI, confidence interval; FEV_1, forced expiratory volume in 1 second; FVC, forced vital capacity; OR, odds ratio.
